# Spatial Inequities in Life Expectancy in Small Areas of Buenos Aires, Argentina 2015–2017

**DOI:** 10.1007/s11524-023-00730-1

**Published:** 2023-05-24

**Authors:** Andrés Trotta, Usama Bilal, Binod Acharya, Harrison Quick, Kari Moore, Serena Mónica Perner, Marcio Alazraqui, Ana Diez Roux

**Affiliations:** Institute of Collective Health, National University of Lanus, Buenos Aires, Argentina; Urban Health Collaborative, Dornsife School of Public Health, Drexel University, Philadelphia, PA, USA; Institute of Collective Health, National University of Lanus, Buenos Aires, Argentina; Urban Health Collaborative, Dornsife School of Public Health, Drexel University, Philadelphia, PA, USA

**Keywords:** Life expectancy, Small area estimation, Urban health, Latin America

## Abstract

Studies of life expectancy (LE) in small areas of cities are relatively common in high-income countries but rare in Latin American countries. Small-area estimation methods can help to describe and quantify inequities in LE between neighborhoods and their predictors. Our objective was to analyze the distribution and spatial patterning of LE across small areas of Ciudad Autónoma de Buenos Aires (CABA), Argentina, and its association with socio-economic characteristics. As part of the SALURBAL project, we used georeferenced death certificates in 2015–2017 for CABA, Argentina. We used a spatial Bayesian Poisson model using the TOPALS method to estimate age- and sex-specific mortality rates. We used life tables to estimate LE at birth. We obtained data on neighborhood socioeconomic characteristics from the 2010 census and analyzed their associations. LE at birth was higher for women (median of across neighborhoods = 81.1 years) compared to men (76.7 years). We found a gap in LE of 9.3 (women) and 14.9 years (men) between areas with the highest and the lowest LE. Better socioeconomic characteristics were associated with higher LE. For example, mean differences in LE at birth in areas with highest versus lowest values of composite SES index were 2.79 years (95% CI: 2.30 to 3.28) in women and 5.61 years (95% CI: 4.98 to 6.24) in men. We found large spatial inequities in LE across neighborhoods of a large city in Latin America, highlighting the importance of place-based policies to address this gap.

## Introduction

More than 80% of the population of Latin America (LA) resides in urban areas, and this urban growth has been especially intense in the last half-century, creating both challenges and opportunities to enact public policies based on scientific findings [1, 2]. LA is also a highly unequal region, with strong residential segregation within countries and cities [3]. The interrelated process of urbanization and segregation directly impact spatial inequities in health outcomes, including mortality and life expectancy. Studies focusing on the heterogeneity of mortality and life expectancy (LE) within urban areas are more common in high-income countries but remain limited to low- and middle-income countries (LMIC) [4–7]. Moreover, much of what is known about health inequities in LA countries is based on city-level indicators [4, 8], not in small areas such as neighborhoods. Challenges for small area estimation (SAE) include the definition and operationalization of what a small area is [9], statistical noise around death counts with small populations [4, 10], and the availability of data, especially given confidentiality concerns [11]. Previous studies found wide inequities in the distribution of LE for sub-city units in LA countries [12, 13]. Although some studies have explored small area variations in LMIC using SAE methods [14, 15], there has been little examination of heterogeneity in LE within the rapidly growing cities of LMIC [16]. For this work, we use the definition of census fraction from the National Statistics Office of Argentina, a unit of analysis that we selected because of the death count required to estimate a reliable small-area estimate and its spatial delimitation used for census data collection.

*Ciudad Autónoma de Buenos Aires* (CABA) is the administrative and political center of Argentina, located in the Buenos Aires metropolitan area, which houses 31.9% of the national population [17]. Significant inequities in social and environmental conditions exist across the city, even though it has a relatively low illiteracy rate, high access to resources, and a lower proportion of the health-uninsured population [18]. Studies on social inequity in CABA have focused on “social maps” describing geographic variation in health and socioeconomic indicators across the city [19, 20] or have characterized multiple dimensions of the sociospatial structure of the city [21]. To our knowledge, no studies have examined variations in LE within CABA using high spatial resolution data to address neighborhood health effects.

To improve on the presence and magnitude of small-area effects on LE, we used state-of-the-art methods to (a) describe the distribution and variability of LE at birth for the period 2015–2017, (b) characterize the spatial patterning of LE across small areas, and (c) analyze its association with socioeconomic characteristics.

## Methods

### Study Setting and Data Sources

This is an ecological study using vital registration data from the 351 *fracciones censales* of CABA in 2015–2017. *Fracciones censales*, henceforth “small areas,” are geostatistical census units based on an average of 5000 households defined by Argentina’s National Institute of Statistics and Census (INDEC). We used georeferenced data from death registry records for CABA obtained from the *Dirección General de Estadística y Censos* (DGEC) for the triennium 2015 to 2017. We obtained population data from the 2010 census and population projections created by DGEC [22] for the 2010 and 2015–2017 periods, given that population distribution from the 2010 census are different from the 2010 projected population distributions (see Appendix 1 for further explanation). We obtained data for socioeconomic data for small areas from the 2010 census [17].

### Mortality and Population

Death records were georeferenced to the small area level by the SALud URBana en America Latina-Urban Health in Latin America (SALURBAL) project, using street address shapefiles created by the *Unidad de Sistemas de Información Geográfica* of the CABA government. Among the total deaths recorded (*n* = 89,410) for the study period, we excluded deaths not georeferenced to the small areas (1,2%), resulting in 88,330 analyzed deaths. Finally, we aggregated death counts by small area, single-year age, and sex for the pooled period of 2015–2017.

To estimate age-specific mortality rates, we needed population denominators by small area, single-year age, and sex for 2015–2017. Because these were not available at that level for that time period, we estimated them using two sources: (a) population counts by small area, single age, and sex from the 2010 census and (b) population projection counts by *comuna* (the next higher administrative level with a median population of 192,677) by 5-year age group and sex, for 2010 and 2015–2017. First, we calculated the census proportion of people for each single-year age/sex/small area within each *comuna* using data from the 2010 census. Second, we graduated the 2010 population projections data by *comuna* using a penalized composite link model (PCLM) [23]. PCLM allows for the graduation (redistribution of a group, in this case, 5-year age groups into single years) of population counts assuming that the age distribution is smooth with limited assumptions [23]. Also, we employed a maximum open-ended age group of 103 years for women and 100 years for men, based on a comparison with the single-year populations available from the 2010 census. We then applied the small-area 2010 census proportions to the population projections to obtain corrected population counts by single-year age/sex/small area for 2010. Lastly, we calculated the proportion of people for each age/sex/small area within each *comuna* based on the 2010 corrected population estimates and applied those proportions to the 2015-2017 population by sex and single age. For more details on population estimation, refer to Appendix 1.

### Socioeconomic Characteristics

Area-level socioeconomic characteristics from the 2010 census were obtained from the SALURBAL project [24]. We selected the following measures for socioeconomic status (SES): percentage of households with water access inside dwellings, percentage of households with overcrowding (more than 3 people per room), percentage of 15–17-year-old population attending school, percentage of the population aged 25 years or above who completed at least high school education, and the unemployment rate. These variables were selected because (a) they have been shown to be associated with LE and mortality in LA cities in previous studies [8, 19] and (b) they are commonly available from census data at the small-area level. Each of these variables was used not as markers per se (patterning of education, overcrowding, water access, etc.) but as markers of the general small area effect (socioeconomic patterning generally). To reduce measurement error in the estimation of socioeconomic status, we also computed a composite index (Z-score) of the aforementioned SES variables. Although there is little agreement on which SES indicator summarizes different aspects of overall health risk better, composite indicators enhance the explanatory power of inequities. A similar index was tested empirically in other studies [25]. To create the composite index, we standardized all variables with a mean of 0 and a standard deviation of 1, and the unemployment and overcrowding variables were reversed (so that higher values signified higher socioeconomic status for all variables). The average of these standardized scores is defined as the composite Z-score representing the socioeconomic characteristics of small areas. A higher score value signifies better living conditions.

### Statistical Analysis

To compute LE, we obtained estimates of single-year age- and sex-specific mortality rates using a Bayesian adaptation of the Tool for Projecting Age-Specific Rates using Linear Splines (TOPALS) method, described in Appendix 2. The city-level mortality schedule served as the standard mortality schedule required in the TOPALS method. To address the unstable log mortality rates of the standard mortality schedule, we performed a LOESS regression, and the smoothed rates were used as the standard schedule for mortality. Based on a Bayesian model, we ran the Markov Chain Monte Carlo algorithm for 100,000. The first 80,000 samples were discarded as *burn-ins*, and the remaining samples were thinned by a factor of 10. We retained 2000 sets of age- and sex-specific mortality rates from the posterior distributions, which were inputted into standard single-age life tables.

We then calculated LE at birth and at ages 20, 40, and 60 years using the DemoTools package in R. The life tables were calculated for each iteration of posterior age-specific mortality estimates, resulting in a total of 2000 life tables with their corresponding life expectancies for each small area. The median of the 2000 samples was reported as the point estimate for LE, while the 2.5th and 97.5th percentiles of the posterior samples were reported as lower and upper credible intervals.

To describe inequities in LE in the city, we calculated the 10th (P10) and 90th percentiles (P90) of the point estimate of LE across small areas by sex and computed the P90–P10 gap. To describe the spatial patterning of LE, we created choropleth maps of the LE using ArcGisPro. To study the association between LE and socioeconomic characteristics, we fit linear regressions of LE on each predictor variable converted into deciles and scored on a continuous scale between 0 and 1. Specifically, for each socioeconomic variable, we assigned the value of 0 if it corresponds to the first decile of its distribution across all small areas. The second decile obtained the value of 1/9, the third decile obtained the value of 2/9, and so on. To acknowledge the uncertainty around the estimates of LE, these models were repeated 2000 times with each set of posterior estimates. Coefficients were pooled using Rubin’s formula [26]. These analyses were done for LE at birth (main results) and LE at ages 20, 40, and 60 years (see Appendix 3 for results). The resulting regression coefficient represents the mean difference in LE in areas with the highest socioeconomic variable (i.e., those in the tenth decile) versus the areas with the lowest value of the socioeconomic variable (those in the first decile) and is presented as the slope index of inequality (SII). Finally, while we accounted for spatial autocorrelation to improve our estimates of LE, we did not account for spatial correlation to describe the predictors of inequities in LE. This was motivated by research from the spatial statistics literature, which recommends against including spatial random effects when the focus is on estimating associations [27] and by the computational burden of fitting our second-stage models 2000 times (once for each set of samples) to account for the uncertainty in the life expectancy estimates.

Analyses were conducted using R® version 4.1.1 software and SAS® 9.4. The data used for this study were aggregated to respect confidentiality and the data agreement policy of the SALURBAL project. None of the sources used had data to identify the individuals involved. The study was approved by the Drexel University institutional review board.

## Results

**Fig. 1:**
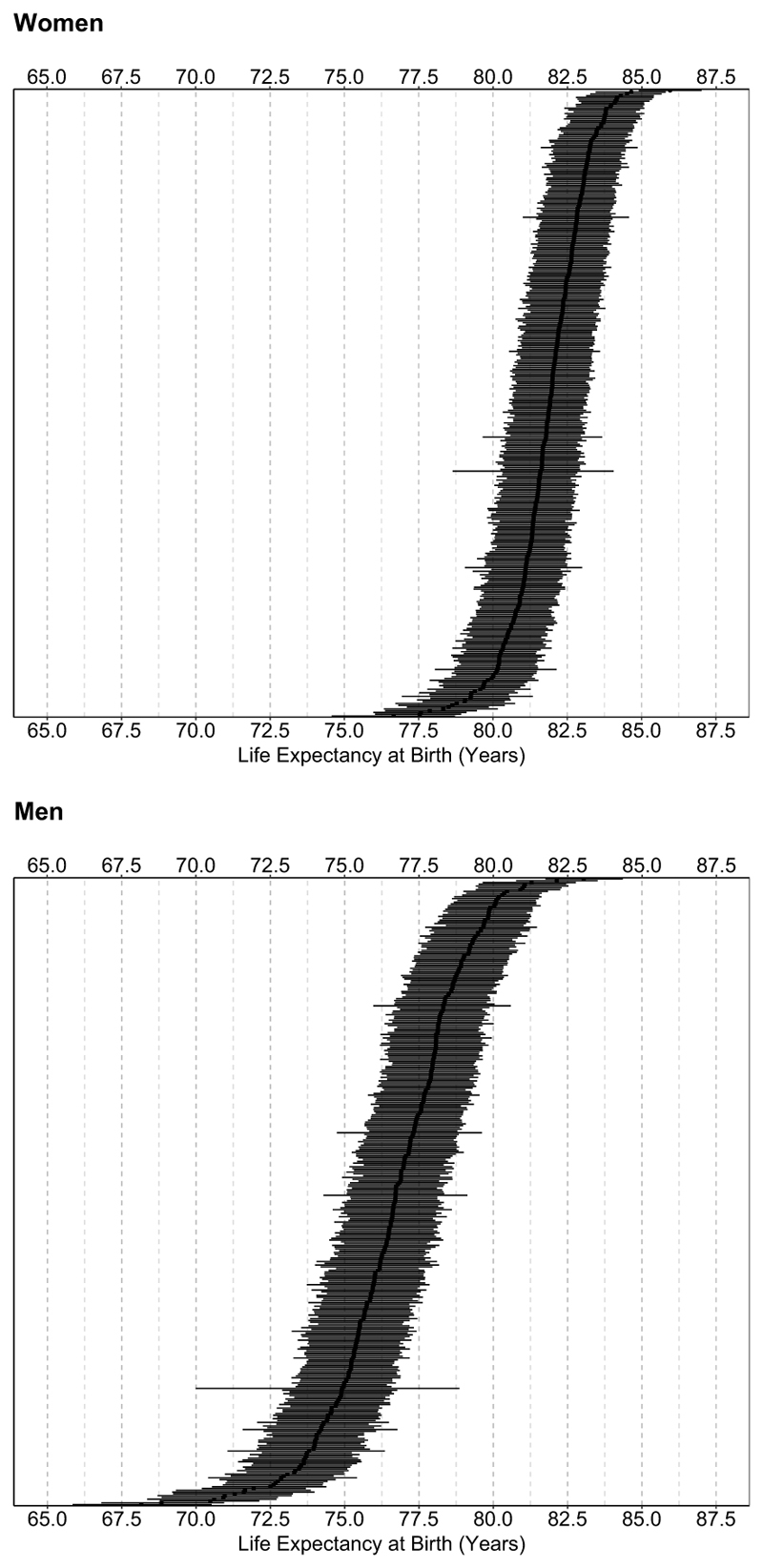
Life expectancy at birth (95% credible intervals) among women and men, by *small area* in Ciudad Autónoma de Buenos Aires, 2015–2017 The vertical (y)-axis represents the small area. Small areas are sorted by the point estimate of LE. The median of the posterior distribution of LE at birth serves as the point estimate of LE, while the 2.5th and 97.5th percentiles of the posterior distribution of LE serve as the lower and upper credible intervals, respectively

Between 2015 and 2017, there were 88,330 deaths in the 351 small areas of CABA. Table 1 describes the socioeconomic characteristics of small areas. Median LE at birth across all small areas was higher for women (81.9 years) compared to men (76.7 years). These patterns held for LE at 20, 40, and 60 years. We found wide variability in LE between small areas, ranging from 76.6 to 85.9 years for women and from 68.1 to 83.0 years for men, demonstrating a gap of 9.3 and 14.9 years between areas with the highest and the lowest LE (Fig. 1). The 90th percentile of LE at birth was 83.2 years for women and 79.3 for men, while the 10th percentile was 80.3 for women and 74.0 years for men, for a P90-P10 difference of 2.9 years and 5.3 years among women and men, respectively.

**Fig. 2:**
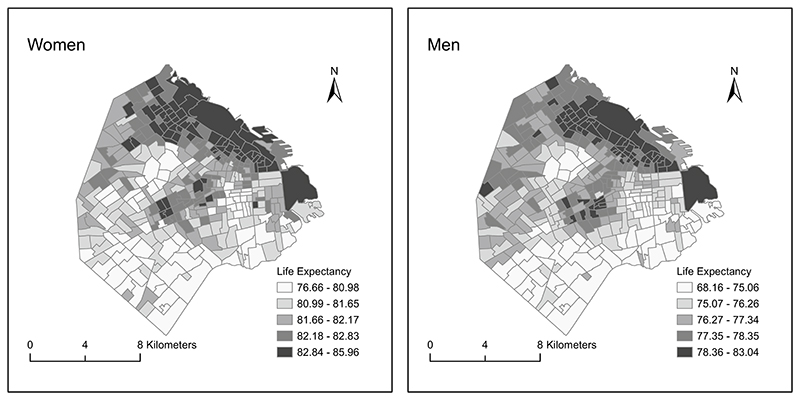
Maps of life expectancy at birth among women and men in small areas of Ciudad Autónoma de Buenos Aires, 2015–2017

We found a general North-South spatial gradient for LE at birth (Fig. 2). LE was higher in the North and North-east parts of the city, as compared to the South and South-western areas (lower LE). In addition, there is a strip in the central region of the city with higher levels of LE, surrounded by other areas of low LE.

**Fig. 3:**
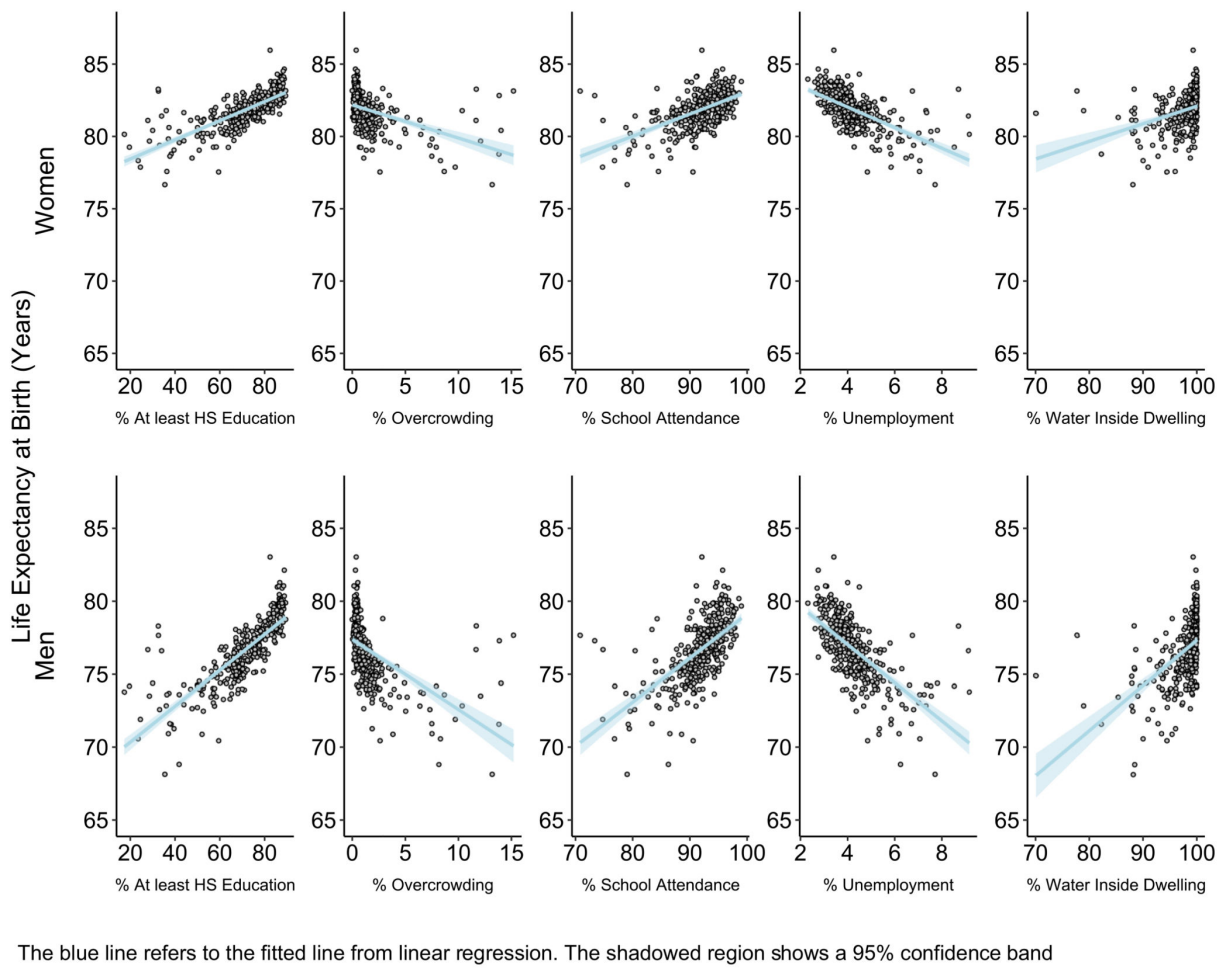
Scatterplots for the association between life expectancy and socioeconomic characteristics by sex for CABA 2015–2017

Figure 3 displays the scatterplots of LE at birth versus small-area socioeconomic characteristics. Education, school attendance, and accessibility to water are positively associated with LE, while unemployment and overcrowding are negatively associated. Table 2 shows the SII in LE at birth associated with each socioeconomic characteristic, by sex. All variables were associated with LE at birth, and the magnitude of association was stronger among men. For example, men living in areas with the highest decile of education (% with at least high school education) had 5.63 years (95% CI: 5.00, 6.25) higher LE at birth than those living in the areas with the lowest levels of education. This association was relatively weaker among women (SII = 2.99 years, 95% CI: 2.51, 3.46). We found a similar pattern with all other socioeconomic variables. For example, men and women living in areas with the highest levels of unemployment had 4.72 and 2.52 years lower LE than those living in areas with the lowest levels of unemployment. The socioeconomic index (composite Z-score) was also strongly associated with LE: small areas at the tenth decile of the Z-score had 5.61 and 2.79 years of higher LE than the areas at the first decile of the Z-score, in men and women, respectively. Analysis with LE at ages 20, 40, and 60 years showed similar results (see Appendix 3).

## Discussion

We found evidence of spatial heterogeneity and intraurban variability in LE at birth and at ages 20, 40, and 60 years in CABA. Overall, LE at birth was 5.2 years higher for women (81.9 vs. 76.7 years in men). We found a P90th–P10th gap of 2.9 and 5.3 years within the city for women and men, respectively. We also evidenced a north-south spatial patterning, with higher LE in the north, and a transitional strip at the core of the city. Furthermore, we found strong associations of socioeconomic status in small areas with LE at birth. Women who lived in small areas with the best socioeconomic indicators can expect to live 2–3 years longer than those living in the most disadvantaged areas, while men who lived in small areas with the better socioeconomic indicators lived 5–6 years longer than those living in the most disadvantaged areas.

Studies focusing on the analysis of spatial variability can provide meaningful information on place-related health effects. Bilal et al. [13] described intraurban variations in LE in six LA cities, including the Buenos Aires metropolitan area (with CABA within), and documented 5.8 and 4.4 years of difference in women’s and men’s LE at birth between the top 90th and bottom 10th percentiles of the metropolitan area of CABA. Sacco et al. [12] reported a significant heterogeneity in LE across departments in the province of Buenos Aires (CABA was not included), showing a 7.6-year gap between departments with the highest and lowest LE at birth. Our findings show that it is possible to reveal and visualize important spatial heterogeneity at a smaller scale within the city. These epidemiological estimates and visualizations can be used to monitor health-based events and to motivate research into the drivers of this spatial heterogeneity.

Although the mechanism underlying the impact of gender inequities in urban agglomerations remains elusive [28], findings from other studies have identified an existing gender gap in life expectancy, and a few have described it for low-income countries [29]. In our study, we found a median age difference of 5.2 years between women and men. Also, the strength of associations between LE and the selected predictors was stronger for men than for women. Previous findings could shed a light on this difference, which could be explained by the concentration of violent deaths among young men [8] and the scale of cities, where larger cities are likely to have a higher proportionate mortality by violence than smaller cities [8, 16, 30]. This gender difference is a health outcome likely to be critical in understanding how cities are shaped by gender inequities that have both biological and nonbiological origins, rooted in gender norms and inequities [29].

The north-south gradient in LE that we observed is similar to that reported by other studies focused on all-cause and cause-specific mortality in Buenos Aires [19]. Our results showed a mosaic pattern in the central part of the city, with a strip of higher LE, like that observed in the northern part of the city. This spatial variability in LE is not random and can be explained by the spatial distribution of people with different socioeconomic characteristics between subcity units (Appendix 4). Sensitivity analyses with LE at ages 20, 40, and 60 showed similar patterns, suggesting that these socioeconomic characteristics are important predictors of mortality outcomes throughout the life span. Unsurprisingly, the spatial patterns of LE exhibit a similar gradient as the spatial pattern observed in the maps of small-area socioeconomic characteristics.

Understanding the historical and economic processes that led to this spatial patterning can help explain inequities in LE. Our findings are consistent with studies inspired by critical geography that proposed habitant-based classificatory typologies for neighborhoods of CABA where the colonial city, the central city, and higher SES residential areas overlap with the northern region—as for this study [21, 31]. In contrast, the southern region of the city was the last area to be incorporated into CABA’s jurisdiction, producing informal settlements and areas of low SES as a result of unplanned interventions [21, 32, 33]. The central strip also corresponds to an area formerly linked to railway employees that has been recently gentrified as part of a renewed city-branding strategy (24).

We also found that small area-level socioeconomic characteristics were strongly predictive of LE. Specifically, advantaged small areas had higher LE, with these associations being stronger among men. These associations could be the result of differences in context (e.g., lower pollution and lower violence in higher SES areas) or in composition (e.g., higher SES individuals in these areas). Differences in composition across areas resulting from residential segregation may explain part of our findings [33]. Our ecological study design cannot differentiate between the effects of segregation patterns and contextual factors.

Quantitative comparisons across studies in the size of inequities are rendered complex by the different areas and metrics used. For example, we used a much smaller spatial unit (*Fracciones censales*) than Bilal et al. (*comuna/partidos*) [13]. The geographical definition of the city can also differ and influence estimates of heterogeneity. We focused on the core city (CABA), while Bilal et al. used a much broader definition based on a larger urban agglomeration that included adjacent areas that are part of the broader metropolitan area [13]. On the one hand, the use of smaller areas may allow us to see more heterogeneity, but the inclusion of larger geographic areas allows comparisons with peripheral areas which, in the LA context, are often more disadvantaged than the core (although these patterns may be rapidly changing with the construction of private neighborhoods in the outskirts of large cities).

This study has several strengths. First, we used data on all deaths registered in CABA during a 3-year period, resulting in more than 88,000 deaths, all georeferenced to a small-area level (average area size = 0.58 km^2^). Second, the Bayesian application of the TOPALS method allowed us to obtain precise and reliable LE estimates. Third, data availability for this study is specifically for the administrative boundaries of CABA. Our findings could inform future research integrating peripheral areas of CABA to describe and study the urban socioeconomic structure [31]. An examination of the larger metropolitan area within which CABA is located would capture a much greater socioeconomic heterogeneity that could potentially exhibit a stronger association between LE and socioeconomic characteristics, as we reported previously for larger geographical areas [13]. Our study also has limitations. First, given that the census data are updated every decade and the 2020 census has not been carried out yet, we relied on the 2010 census for SES variables. The time difference between census (2010) and georeferenced deaths (2015–2017) could bias these results. We also relied on the 2010 population along with population projections for 2015–2017 to obtain population denominators. The total city population has only changed by 2% over the 7-year period, according to previous projections, which would allow us to assume that the impact is likely to be small [22]. Second, deaths that could not be georeferenced were less than 1.5% each year for all age groups and 6% for infants (under 1 year). This lack of georeferenced data could be related to areas of low socioeconomic characteristics, whose residents are less likely to report vital events than residents in areas with high socioeconomic status [34]. Consequently, LE estimates could be overestimated in lower socioeconomic areas, resulting in a conservative estimation of the associations between area socioeconomic status and LE.

In summary, we found wide within-city spatial and socioeconomic heterogeneities in LE across small areas of one of the wealthiest cities of Argentina, a middle-high-income LA country. These heterogeneities are driven by modifiable factors that configure inequities that could potentially be addressed through oriented place-based policies, for example, community gender-based interventions.

## Supplementary Material

Appendix

## Figures and Tables

**Table 1:** Number of deaths, life expectancy among women and men, and selected sociodemographic characteristics by *small area* (*n* = 351). Ciudad Autónoma de Buenos Aires, 2015-2017

Characteristics	Median (10th–90th percentile)	
	Women	Men
Number of deaths	140 (96, 191)	110 (74, 156)
Estimated population^a^	13,628 (10,030, 18,082)	11,715 (8,530, 16,203)
Life expectancy at birth^a^	81.9 (80.3, 83.2)	76.7 (74.0, 79.3)
Life expectancy at age 20^a^	62.6 (60.9, 63.9)	57.5 (54.7, 60.0)
Life expectancy at age 40^a^	43.0 (41.5, 44.3)	38.2 (35.9, 40.6)
Life expectancy at age 60^a^	24.6 (23.4, 25.6)	20.5 (18.8, 22.3)
Population aged 15 or younger^a^ (%)	17.7 (13.1, 23.0)	21.7 (17.0, 27.0)
Population aged 65 or older^a^ (%)	19.8 (15.7, 24.0)	13.2 (10.4, 17.1)
Households with water inside dwellings^b^ (%)	99.1 (93.5,99.9)	
Households with overcrowding^b^ (%)	0.7 (0.25, 2.9)	
School attendance among 15-17 years old^b^ (%)	99.2 (86.3, 96.3)	
At least high school education^b^ (%)	72.7 (52.7, 86.7)	
Unemployment^b^ (%)	4.1 (3.1, 5.5)	

Overcrowding: proportion of households with more than 3 people per room. Unemployment: proportion among the population 15 years or above in the labor force

a Data from 2015 to 2017

b Data from the 2010 census

**Table 2:** Slope index of inequality (SII) in life expectancy at birth (years) associated with small-area characteristics in Ciudad Autónoma de Buenos Aires, 2015-2017

Variable	SII in LE at birth in years (95%confidence intervals)	
	Women	Men
At least high school education (%)	2.99 (2.51, 3.46)	5.63 (5.00, 6.25)
Households with overcrowding (%)	−2.19 (−2.71, −1.68)	−4.77 (−5.47, −4.08)
School attendance among 15–17 years old (%)	2.15 (1.64, 2.65)	4.48 (3.77, 5.18)
Unemployment (%)	−2.52 (−3.01, −2.04)	−4.72 (−5.40, −4.03)
Households with water inside dwellings (%)	1.93 (1.41, 2.45)	4.38 (3.66, 5.09)
Composite Z-score	2.79 (2.30, 3.28)	5.61 (4.98, 6.24)

The models were run in a univariate fashion, one variable at a time. Small-area characteristics were transformed into deciles. The SII represents the mean difference in life expectancy in areas with the highest predictor variable (i.e., those in the tenth decile, having value = 1) versus the areas with the lowest value of the predictor variable (those in the first decile, having value = 0). Socioeconomic data for small areas came from the 2010 census. Overcrowding: proportion of households with more than three people per room
