# Supplementary material for: Spatial Inequities in Life Expectancy in Small Areas of Buenos Aires, Argentina 2015–2017
Source: J Urban Health. Author manuscript; Available in PMC 2023 Jul 10. (PMC10323071; doi:10.1007/s11524-023-00730-1)
Supplement: Appendix [file EMS176984-supplement-Appendix.pdf]

Appendix 1

Estimating the Corrected Population for 2010

Although the 2010 census provides population counts by single-year age (0,1,…,110), sex, and small area, the population distribution from the 2010 census is different from the 2010 projected population distribution. In particular, the 2010 projected population makes a correction for the census that omitted an estimated 5.13% of the population of Buenos Aires, most of which impacted the younger age groups. This omission was determined by using prior censuses, vital statistics, and migration registration (from the Direccion General de Estadistica y Censos, Gobierno de la Ciudad de Buenos Aires). However, the 2010 projected population is available at the comuna level, with the highest age group being 80 years. We obtained corrected 2010 population estimates by single age (0,1,..85), sex, and small area as follows:

- Aggregated the census 2010 population into single-year ages (0,1,… 85+) by sex and small area. Population aged ≥ 85 years are included in the highest age category, 85+. All other ages remain as a single year.
- Graduated the projected 2010 population for each comuna by sex into single ages using the penalized composite link model (PCLM)[1]. This redistributes the population into single-year age groups. Population aged ≥ 85 years are included in the highest age category, 85+, to match the census population.
- Using the census 2010 data, we calculated the proportion of the comuna population that lives within a given small area for each age–sex combination.

Applied those proportions to the projected 2010 population to obtain the corrected age and sex counts for each small area for 2010.

Let $n_{asi;\,j}$ represent the 2010 census population for age (a), sex (s), and small areas (i). Let the small area (i) be nested within the comuna (j). Assume there are N_i small areas nested in the jth comuna. Then, the proportion of people living in each small area of a given comuna (i.e., the proportion of the comuna population within the small area for each sex–age combination) is,

$$r_{asi;j} = \frac{n_{asi;j}}{\sum_{i=1}^{i=Ni} n_{asi;j}}$$

Now, let P_{asj} be the projected 2010 population for a, s, and j.

The corrected 2010 population estimate by age, sex, and L2.5 will be: $E_{asi;\,j} = P_{asj} \times r_{asi;\,j}$.

Estimating the Population for 2015–2017—Was Used as the Denominator

We estimate the population counts by single-year age, sex, and a small area for 2015–2017—the years that correspond to the mortality data—with the following steps.

- We graduate the 2015–2017 comuna-level projected population by sex into single ages.
- We calculate the proportion of people in each small area out of comuna-level population by age and sex based on the 2010 corrected population estimates
- We apply those proportions to the 2015–2017 population by sex and single age

For example, if $P_{2015\ asj}$ is the 2015 comuna-level projected population at jth comuna, for a and s, and if $E_{asi;\,j}$ is the estimated 2010 population for the small area calculated above, the estimated population for the year 2015 by age, sex, and a small area will be:

$$E_{2015\ asi;j} = P_{2015\ asj} \times \frac{E_{asi;j}}{\sum_{i=1}^{i=Ni} E_{asi;j}}$$

[1] The maximum open-ended age group in population graduation was set at 103 for females and 100 for males.

Appendix 2

Modeling Mortality Rates

In this paper, we employ the Bayesian adaptation of the tool for projecting age patterns using the linear spline method (TOPALS), which incorporates spatial smoothing in small areas [4].

For a $\{a=0,1,2,\ldots,85+\}$ and i $\{i=1,2,\ldots,351\}$ with corresponding population (n_{ia}), we assume that the number of deaths comes from a Poisson distribution with an underlying rate (λ_{ia}).

$$y_{ia} \mid \lambda_{ia} \sim \text{Pois}\left(n_{ia}\,\lambda_{ia}\right)$$

We further assume that the vector of log mortality rates in i is,

$$\log\left(\boldsymbol{\lambda}_{i.}\right) = \log\left(\boldsymbol{\lambda}^*\right) + \boldsymbol{B}\,\boldsymbol{\beta}_{\boldsymbol{k}.},$$

where $\boldsymbol{\lambda}_{\boldsymbol{i}}$ is the vector with elements λ_{ia} representing age-specific mortality rates in small areas, $\boldsymbol{\lambda}^*$ is the vector of the standard mortality schedule (i.e., the smoothed city-level rates), $\boldsymbol{B}$ is a matrix of constants of size 86 × 7 in which each column is a linear B-spline basis function ($\boldsymbol{\beta}_{\boldsymbol{k}}$) is a vector of parameters with elements β_{ik} representing offsets to the standard schedule. We used the 2015–2017 mortality schedule for the whole city of CABA as the standard schedule. We obtained a smoothed version of this schedule by fitting a LOESS regression of log(mortality) on age.

We define knots at ages $t_0,\ldots,t_6$ = (0, 1, 10, 20, 40, 70, 85). For ages a in {0, 1, 2, ..., 85} and columns k in {0,...,6}, the basis functions in $\boldsymbol{B}$ are:

$$\frac{a - t_{k-1}}{t_k - t_{k-1}}\ \textit{if}\ t_{k-1} \le a \le t_k;$$

$$\frac{t_{k+1} - a}{t_{k+1} - t_k}\ \textit{if}\ t_k \le a \le t_{k+1};$$

$$0\ \textit{otherwise}.$$

We further decompose the β_{ik} into the intercepts at each knot β_{0k}, the spatial random effects (z_{ik})and unstructured random effects (ϕ_{ik}) that vary by knot age and area.

$$\beta_{ik} = \beta_{0k} + z_{ik} + \phi_{ik}.$$

We assign the unstructured, non-spatial random effect (ϕ_{ik}) an exchangeable zero-mean normal prior to the knot-specific variance ($\sigma^2_{ns;k}$). The variance parameter in turn receives the uninformative inverse gamma hyperprior with the shape and rate parameters of 0.05 and 0.005, respectively.

That is,

$$\phi_{ik} \sim \text{Normal}\left(0, \sigma^2_{ns;k}\right)$$

$$\sigma^2_{ns;k} \sim \text{Inverse Gamma}\ (0.05, 0.005)$$

For the intercept (β_{0k}), we assign a vague normal prior with a mean of 0 and variance of 1000. That is,

$$\beta_{0k} \sim \text{Normal}\ (0, 1000)$$

For the spatial random effect (z_{ik}), we assign the intrinsic conditional autoregressive (ICAR) prior distribution for each knot, k. We define areas i and j as neighbors if they share one or more common vertex between boundaries, commonly referred to as Queen's contiguity.

For any given knot and for each area, the conditional expected value of z_{ik} given the remaining values are the mean of its neighboring areas and the variance of z_{ik} is inversely proportional to the number of neighbors in that area, m_i.

If we drop the subscript for knot here, we can denote the CAR distribution as:

$$z_i \mid z_{-i}, \boldsymbol{W},\ \sigma^2_z \sim \text{Normal}\left(\bar{z}_i, \frac{\sigma^2_z}{m_i}\right),$$

where

$$\bar{z}_i = \sum_{j, j\neq i} \frac{w_{i,j} z_k}{m_i}$$

Here, $\boldsymbol{W}=[w_{i,\,j}]$ is 351 ×351 adjacency matrix with elements $w_{i,\,j}=1$ if areas i and j are neighbors and 0 otherwise. We complete the prior specification by assigning an uninformative inverse gamma prior (0.001, 0.001) for the variance of CAR random effects.

The models were run using WinBUGS by calling the software with the R2WinBUGS package in R for 50,000 iterations for each of the two chains. For each chain, the first 40,000 samples were discarded as burn-ins, and the remaining samples were thinned by a factor of 10 to reduce the autocorrelation of the samples. We fit the models for males and females separately.

Appendix 3

Table 3 Slope index of inequality (95% CI) in life expectancy (years) at ages 20, 40, and 60 associated with small area characteristics in CABA

Variable	At 20		At 40		At 60	
	Women	Men	Women	Men	Women	Men
At least high school education (%)	2.94 (2.48, 3.40)	5.46 (4.86, 6.06)	2.72 (2.27, 3.16)	4.76 (4.21, 5.32)	1.99 (1.59, 2.39)	3.40 (2.93, 3.87)
Households with overcrowding (%)	−2.14 (−2.64, −1.64)	−4.60 (−5.27, −3.93)	−1.97 (−2.45, −1.49)	−3.92 (−4.54, −3.31)	−1.42 (−1.84, −1.00)	−2.73 (−3.24, −2.21)
School attendance among 15–17 years old (%)	2.10 (1.60, 2.59)	4.33 (3.64, 5.01)	1.92 (1.44, 2.39)	3.68 (3.05, 4.31)	1.37 (0.96, 1.78)	2.54 (2.02, 3.06)
Unemployment (%)	−2.48 (−2.95, −2.01)	−4.58 (−5.24, −3.91)	−2.29 (−2.75, −1.83)	−3.95 (−4.56, −3.35)	−1.68 (−2.08, −1.28)	−2.76 (−3.27, −2.25)
Households with water inside dwellings (%)	1.88 (1.37, 2.39)	4.22 (3.52, 4.91)	1.74 (1.25, 2.23)	3.63 (2.99, 4.26)	1.26 (0.84, 1.69)	2.57 (2.04, 3.09)
Composite Z-score	2.73 (2.26, 3.21)	5.43 (4.82, 6.03)	2.52 (2.06, 2.97)	4.69 (4.13, 5.25)	1.83 (1.42, 2.24)	3.29 (2.81, 3.78)

The models were run in a univariate fashion, one variable at a time. Small-area characteristics were transformed into deciles. The SII represents the mean difference in life expectancy in areas with the highest predictor variable (i.e., those in the tenth decile, having value = 1) versus the areas with the lowest value of the predictor variable (those in the first decile, having value = 0. Socioeconomic data for small areas came from the 2010 census; overcrowding: proportion of households with more than three people per room

Appendix 4

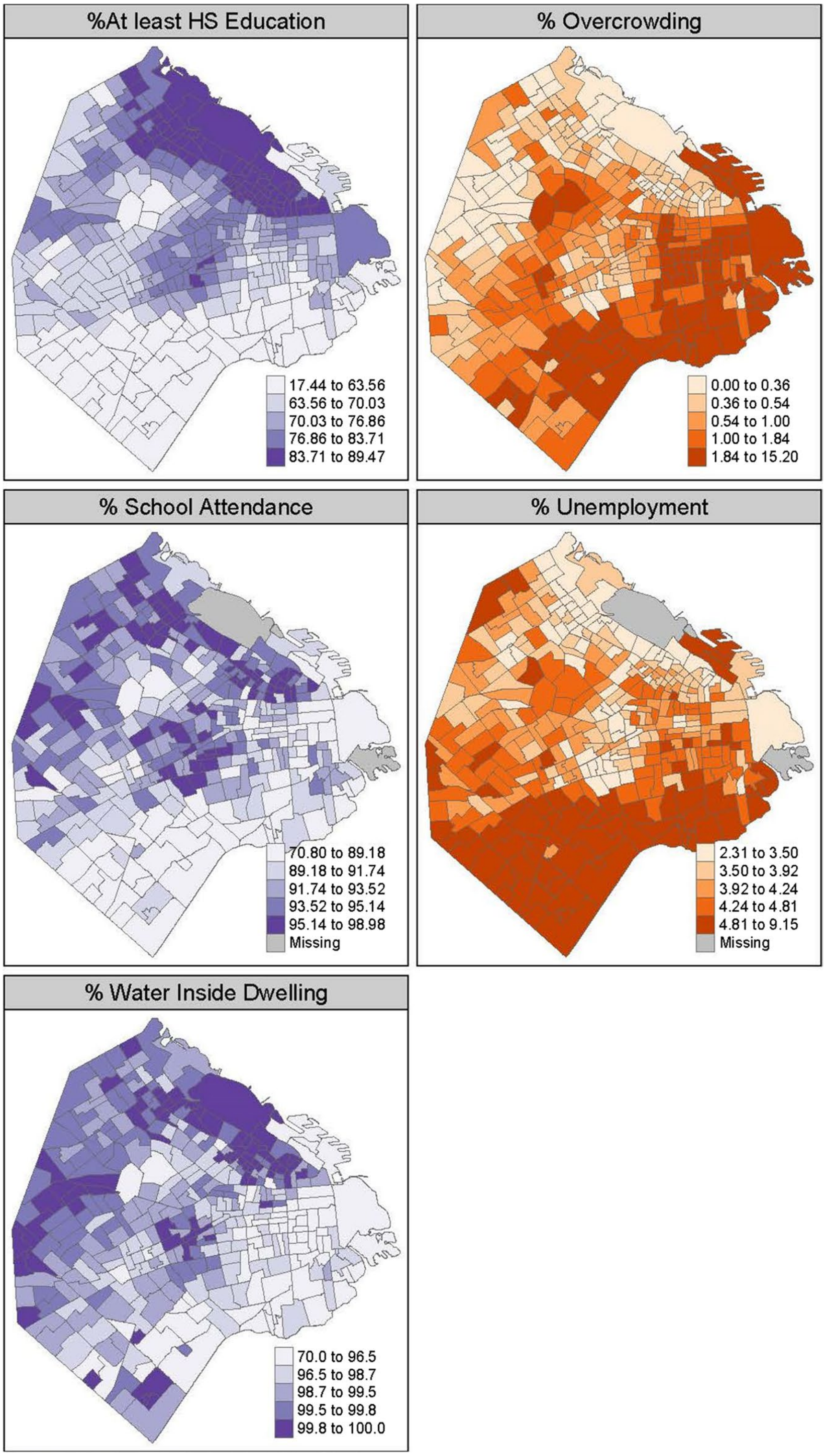

Fig. 4 Choropleth maps of selected socioeconomic characteristics in small areas of CABA. Map cut-offs are based on quintiles; on the purple scale maps, a higher percentage indicates better socioeconomic characteristics of the small areas; on the orange scale maps, a higher percentage indicates a worse socioeconomic characteristic of the small areas; data on socioeconomic characteristics are from the 2010 Argentina census
